# Use of proton pump inhibitors after laparoscopic gastric bypass and sleeve gastrectomy: a nationwide register-based cohort study

**DOI:** 10.1038/s41366-024-01593-5

**Published:** 2024-07-23

**Authors:** Johanne Gormsen, Jonas Sanberg, Ismail Gögenur, Frederik Helgstrand

**Affiliations:** 1 Center for Surgical Science, Department of Surgery, Zealand University Hospital, Køge, Denmark; 2 Upper GI and HPB Section, Department of Surgery, Odense University Hospital, Odense, Denmark

**Keywords:** Epidemiology, Stomach diseases, Disease prevention

## Abstract

**Background/Objectives:**

L-RYGB and L-SG are the dominant bariatric procedures worldwide. While L-RYGB is an effective treatment of coexisting gastroesophageal reflux disease (GERD), L-SG is associated with an increased risk of de-novo or worsening of GERD. The study aimed to evaluate the long-term use of proton pump inhibitors (PPI) following laparoscopic Roux-en-Y gastric bypass (L-RYGB) and sleeve gastrectomy (L-SG).

**Subjects/Methods:**

This nationwide register-based study included all patients undergoing L-RYGB or L-SG in Denmark between 2008 and 2018. In total, 17,740 patients were included in the study, with 16,096 and 1671 undergoing L-RYGB and L-SG, respectively. The median follow up was 11 years after L-RYGB and 4 years after L-SG. Data were collected through Danish nationwide health registries. The development in PPI use was assessed through postoperative redeemed prescriptions. GERD development was defined by a relevant diagnosis code associated with gastroscopy, 24 h pH measurement, revisional surgery or anti-reflux surgery. The risk of initiation of PPI treatment or GERD diagnosis was evaluated using Kaplan–Meier plots and COX regression models. The risk of continuous PPI treatment was examined using logistic regression modeling.

**Results:**

The risk of initiating PPI treatment was significantly higher after L-SG compared with L-RYGB (HR 7.06, 95% CI 6.42–7.77, *p* < 0.0001). The risk of continuous PPI treatment was likewise significantly higher after L-SG (OR 1.45, 95% CI 1.36–1.54, *p* < 0.0001). The utilization of PPI consistently increased after both procedures. The risk of GERD diagnosis was also significantly higher after L-SG compared with L-RYGB (HR 1.93, 95% CI 1.27–2.93, *p* < 0.0001).

**Conclusions:**

The risk of initiating and continuing PPI treatment was significantly higher after L-SG compared with L-RYGB, and a continuous increase in the utilization of PPI was observed after both procedures.

## Introduction

Gastroesophageal reflux disease (GERD) is a multifactorial condition characterized by bothersome symptoms resulting from regurgitation of gastric contents into the esophagus [1]. GERD adversely impacts the health-related quality of life and is linked to an elevated risk of esophagitis, esophageal strictures, Barrett’s esophagus and esophageal adenocarcinoma [2]. Initial treatment involves lifestyle modifications, followed by proton pump inhibitors (PPI) if these modifications prove ineffective. For cases refractory to pharmacological treatment, anti-reflux surgery is considered a standard of care [3]. Patients with coexisting severe obesity are not suitable candidates for conventional anti-reflux surgery. Instead, bariatric surgery is the preferred strategy in case of GERD and severe obesity [4]. Bariatric surgery is recognized as the most effective intervention for severe obesity, leading to sustained weight loss and remission of obesity-related comorbidities including GERD [5]. While laparoscopic Roux-en-Y gastric bypass (L-RYGB) has traditionally been regarded as the gold standard, laparoscopic sleeve gastrectomy (L-SG) has recently become the dominant bariatric procedure in the United States and Europe [6, 7]. In case of GERD and severe obesity, The European Association of Endoscopic Surgery (EAES) guidelines recommend L-SG with sphincter and hiatal repair for moderate cases, while L-RYGB is recommended for severe cases [4, 8]. Contradictory, a notable incidence of de-novo GERD has been reported after bariatric surgery, particularly following L-SG [9]. The lower risk for GERD after L-RYGB is attributed to reduced intragastric pressure, combined with a smaller gastric reservoir and fewer parietal cells [10]. Studies investigating the pathophysiological mechanisms of GERD after L-SG, associated symptoms with increased intragastric pressure due to the low-volume and low-compliance sleeve conduit [11, 12]. Other hypotheses explaining the increased incidence of GERD after L-SG include alterations in the esophageal motility, widening of Hiss angle and the development of hiatal hernia [13, 14]. If symptoms persist despite lifestyle interventions and use of PPI, revisional surgery to L-RYGB has proven effective in treating GERD after L-SG [15]. This study aimed aim to evaluate the development in PPI use and secondarily the risk of GERD diagnosis after L-RYGB and L-SG.

## Methods

### Setting

This nationwide register-based study included patients undergoing either L-RYGB or L-SG in Denmark between 2008 and 2018. In Denmark, health care including bariatric surgery is funded through taxation for all citizens. According to national Danish guidelines from 2010, bariatric surgery was recommended for individuals with a Body Mass Index (BMI) ≥ 35 and an obesity-related comorbidity (including poorly regulated type 2 diabetes or hypertension, infertility, obstructive sleep apnea or osteoarthritis of the hips or knees). In 2017, the national guidelines were expanded to encompass patients with an individually assessed significant risk of developing obesity-related comorbidities [16]. Monosymptomatic reflux is not considered an indication for bariatric surgery in Denmark.

### Data sources and variables

Prospective data collection was conducted through the Danish national health registries, utilizing the following sources for nationwide data:

1. Danish Obesity Surgery Registry (DOSR) [17]: A national clinical registry under the Danish Clinical Quality Program (RKKP) systematically registering all patients undergoing bariatric surgery at public or private institutions since 2010. Overseeing by a national steering committee ensures the quality of registrations. DOSR provided preoperative data on BMI, smoking, comorbidities and intraoperative factors.
2. Danish National Patient Register (NPR) [18]: Containing in-hospital diagnoses since 1977 and outpatient diagnoses since 1994, the NPR was used to identify postoperative diagnoses with GERD, endoscopies, 24-hour pH measurements, anti-reflux or revisional surgery. Preoperative diagnosis codes with a lookback period of five years were used for calculation of Charlson Comorbidity Index (CCI) [19]. The NPR was restructured in 2019 and the new validated research-oriented data model was launched in 2022.
3. Danish National Prescription Register [20]: Utilized for identification of pre- and postoperative redeemed prescriptions of PPI and ulcerogenic pharmaceuticals.
4. Civil Registration System [21]: Providing information on death and emigration during the follow up period.
5. Statistics Denmark [22]: Providing data on socioeconomic factors including marital status, occupational status and educational level.

### Study population

The study population encompassed all adults (age 18+) undergoing L-RYGB or L-SG at a private or public hospital in Denmark from January 1, 2008 to December 31, 2018. The index date was defined as the date of L-RYGB or L-SG. Population identification relied on NOMESCO surgery codes JDF11, JDF41 and JDF97 from DOSR and NPR [17, 18]. In case of discrepancies between DOSR and NPR regarding the procedure type, DOSR registrations took precedence. For discrepancies related to the surgery date, the NPR registrations were considered valid. Only the first procedure for patients undergoing revisional surgery was included. Patients were excluded in case of preoperative GERD diagnosis (diagnosis codes K209B, K21* (* indicating all related diagnoses)) related to a gastroscopy or 24 h pH measurement (procedure codes UJD02, UJD05, ZZ1007, ZZ1007A, ZZ1009) or a bariatric surgery code before the index date (surgery codes JDF10-11, JDF20-21, JDF40-42, JDF50-51, JDF96-98). All Danish residents are registered with a unique civil registration number, which makes it possible to combine data from multiple registries and achieve complete follow up [23]. Only patients with a valid civil registration number were included.

### Definitions

Initiation of PPI treatment (Anatomical Therapeutic Chemical (ATC) codes A02BC*) was defined by ≥2 redemptions within one year, with the index date being the date of the first redeemed prescription. Dosages were computed based on omeprazole-equivalent dosages [24] and reported as defined daily doses (DDD), considering one DDD as 20 mg omeprazole-equivalent. Continuous PPI treatment was defined by an average PPI dose of 20 mg omeprazole-equivalent throughout a year after the first postoperative year combined with persistent PPI use at the final year of follow up. Ulcerogenic pharmaceuticals encompassed non-steroidal anti-inflammatory drugs and anticoagulants including acetylsalicylic acid (ATC codes B01A-C*, B01AE-AX*, M01AA-AH*, M01BA*). Treatment initiation was defined by ≥2 redemptions within one year.

A previously used algorithm for definition of GERD from NPR was revised with regard to previous bariatric surgery [25]. The algorithm was revised through an exploratory review of the data. Patients were defined as having GERD if they met at least one of the following criteria:

1. A diagnosis code indicating GERD (K209B, K21*) linked with a gastroscopy (UJD02, UJD05). The permissible timeframe from diagnosis to gastroscopy was six months before and 12 months after.
2. A diagnosis code indicating GERD (K209B, K21*) linked with 24 h pH measurement (ZZ1007, ZZ1007A, ZZ1009).
3. A diagnosis code indicating GERD (K209B, K21*) linked with revisional surgery to L-RYGB or conversion of RYGB (JDF10-11, JDF50-51).
4. Undergoing anti-reflux surgery postoperatively (JBC00-02, JBW96-98).

### Ethics approval and consent to participate

The research was in accordance with the Declaration of Helsinki. The study was approved by the Danish Data Protection Agency (no. REG-055-2021). Approval for pharmaceutical data was granted by the Danish Health Data Authority (no. FSEID-00005705). Data access was approved and maintained by Statistics Denmark (no. 708334). Informed consent and approval from the Danish Patient Safety Authority or the Committee on Health Research Ethics was not required, given the absence of medical records acquisition or intervention.

### Statistical analyses

Categorical data were presented by proportions and percentages. Continuous data were examined by plotting of histograms and q-q plots and presented with mean or median and standard deviation (SD) or interquartile range (IQR). Differences were assessed using the chi-square test and Student’s *t* test. PPI use was depicted as the mean DDD of omeprazole-equivalents prescribed per year, with differentiation based on type of bariatric surgery and preoperative PPI treatment. The Kaplan–Meier estimator and Log-Rank test were used for assessment of the risk of PPI initiation or GERD diagnosis. Cox regression models were utilized for both outcomes, adjusting for sex, age, CCI, preoperative PPI use and postoperative diagnosis of gastroenteric ulcer. A subgroup analysis was performed including BMI, smoking, marital status, occupational status and educational level. The included variables were based on clinical relevance. Survival statistics intervals commenced at the index date until the end of follow-up (31 December 2021) or until PPI initiation or GERD diagnosis. Patients were censored in case of death or emigration. Hazard ratios (HR) with 95% confidence intervals (95% CI) were reported. The risk of continuous PPI treatment was examined using logistic regression modeling, adjusting for sex, age, CCI, preoperative PPI use and postoperative diagnosis of gastroenteric ulcer. A subgroup analysis was performed including BMI, smoking, marital status, occupational status and educational level. Odds ratios (OR) with 95% CI were reported. Two-sided *p*-values < 0.05 were considered statistically significant. Patients with missing data were excluded from the analyses. Statistical analyses were conducted using SAS, version 9.4 (SAS Institute Inc) and plots were generated using R version 4.2.3 (R Foundation for Statistical Computing, Vienna, Austria). Reporting adheres to the STROBE (Strengthening of the Reporting of Observation Studies in Epidemiology) guidelines [26].

## Results

**Fig. 1:**
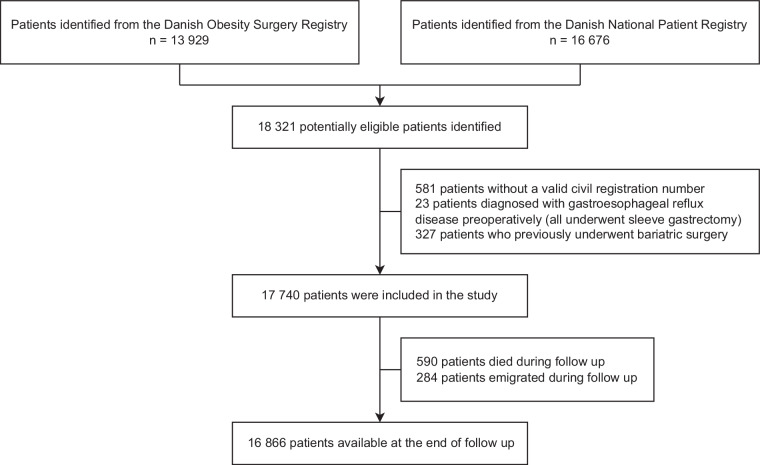
Flow diagram.

A total of 17,740 patients were included in the study. Among the 16,069 patients undergoing L-RYGB, the median follow-up was 11 years (IQR 8–12), while the 1671 patients undergoing L-SG were followed for a median of 4 years (IQR 3–5). At the end of the study, 16,866 (95%) patients were available for follow up (see Fig. 1 for details). Patients undergoing L-RYGB were significantly younger, more likely to smoke, had a higher BMI and had a higher prevalence of type 2 diabetes. However, they had a significantly lower CCI. A significantly larger proportion of patients undergoing L-SG were preoperatively treated with PPI compared with patients undergoing L-RYGB (6% and 2%, respectively; *p* < 0.0001). Moreover, a higher percentage of patients undergoing L-RYGB were employed, albeit with lower educational level, when compared with patients undergoing L-SG. See Table 1 for baseline patient characteristics.

**Table 1:** Baseline characteristics.

	Laparoscopic gastric bypass *n* = 16,069	Laparoscopic sleeve gastrectomy *n* = 1671	*P* value
Sex ratio (M:F)	3908:12,161 (24.3:75.7)	425:1246 (25.4:74.6)	0.313
Age (years)			<0.0001
<30	2213 (13.8)	202 (12.1)	
30–39	5188 (32.3)	454 (27.2)	
40–49	5202 (32.4)	593 (35.5)	
50–59	2942 (18.3)	343 (20.5)	
>60	524 (3.3)	79 (4.7)	
BMI (kg/m^2^) *n* = 13,552			<0.0001
<39.9	2218 (18.6)	461 (28.2)	
40–44.9	4465 (37.5)	502 (30.7)	
45–49.9	2884 (24.2)	333 (20.4)	
50–54.9	1511 (12.7)	183 (11.2)	
>60	840 (7.1)	155 (9.5)	
Smoking status *n* = 13,233			<0.0001
Yes	2584 (22.2)	259 (16.1)	
Previously	2996 (25.8)	500 (31.1)	
Never	6046 (52.0)	848 (52.8)	
Charlson comorbidity index			0.001
0	11,589 (72.1)	1150 (68.8)	
1	2782 (17.3)	305 (18.3)	
2	740 (4.6)	111 (6.6)	
≥3	958 (6.0)	105 (6.3)	
Comorbidities			
Type 2 diabetes	3580 (22.3)	341 (20.4)	0.079
Insulin dependent	519 (14.5)	36 (10.6)	
Non-insulin dependent	3061 (85.5)	305 (89.4)	
Hypertension	3781 (23.5)	479 (28.7)	<0.0001
Cardiovascular disease	440 (2.7)	51 (3.1)	0.457
Liver disease	147 (0.91)	27 (1.6)	0.006
Renal disease	106 (0.66)	32 (1.9)	<0.0001
Pulmonary disease	1782 (11.1)	253 (15.1)	<0.0001
Obstructive sleep apnea	1977 (12.3)	364 (21.8)	<0.0001
Preoperative treatment with PPI	290 (1.80)	95 (5.69)	<0.0001
Marital status *n* = 17,618			0.753
Married or cohabiting	10,717 (67.1)	1107 (66.8)	
Single	5243 (32.9)	551 (33.2)	
Occupational status *n* = 17,726			0.001
Employed	12,048 (75.0)	1188 (71.2)	
Unemployed	2408 (15.0)	309 (18.5)	
Retired	1602 (10.0)	171 (10.3)	
Educational level *n* = 17,433			<0.0001
Primary (up to 10th grade)	5204 (33.0)	384 (23.4)	
High-school and vocational education	7636 (48.4)	781 (47.5)	
Short higher education, bachelor and equivalent	2713 (17.2)	416 (25.3)	
Master or equivalent including PhD grade	237 (1.5)	62 (3.8)	

Values are *n* (%) unless otherwise indicated.

*M* Male, *F* Female, *BMI* Body Mass Index, *PPI* proton pump inhibitors.

### Postoperative initiation of PPI treatment

**Fig. 2:**
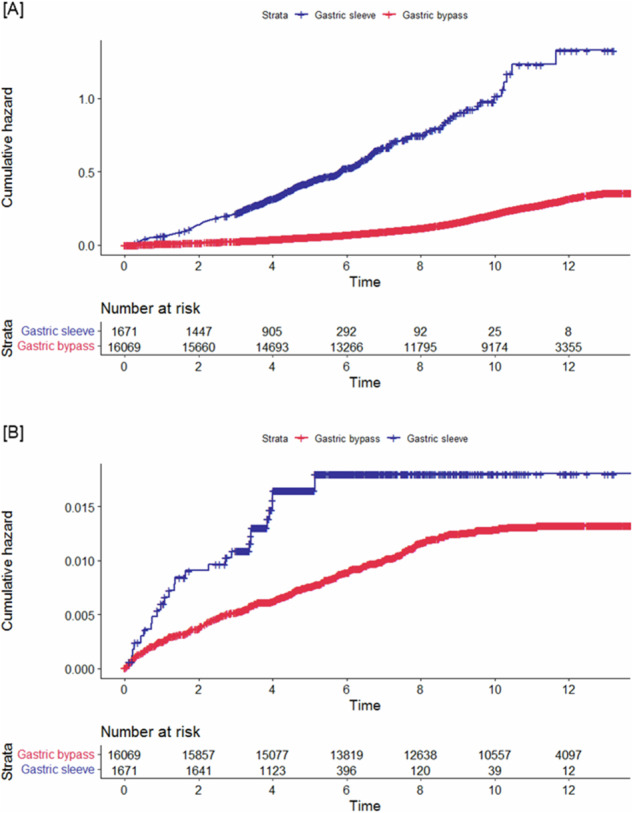
Kaplan Meier estimates of proton pump inhibitor initiation and diagnosis with gastroesophageal reflux disease. Risk of [**A**] starting treatment with proton pump inhibitors and [**B**] being diagnosed with gastroesophageal reflux disease after laparoscopic Roux-en-Y gastric bypass and sleeve gastrectomy. Cumulative hazard (%); Time (years).

After L-SG, 37% of the patients commenced PPI, in contrast to 21% of the patients undergoing L-RYGB. The risk of initiating PPI treatment was significantly elevated after L-SG compared with L-RYGB (*p* < 0.0001). The risk of initiating PPI treatment over time is depicted in Fig. 2A. In a multivariate Cox regression model adjusted for sex, age, preoperative CCI, preoperative PPI treatment and postoperative occurrence of gastroenteric ulcer, the heightened risk after L-SG persisted, HR 7.06 (95% CI 6.42–7.77). In a subgroup analysis including 11,494 patients furtherly adjusted for BMI, smoking, marital status, occupational status and educational level, the heightened risk among L-SG patients persisted, HR 5.74 (95% CI 5.17–6.39) (see Supplementary Content 1). Risk factors for initiation of PPI treatment additionally included female sex, higher age, smoking, higher CCI, unemployment or retirement, preoperative PPI treatment and incidence of postoperative gastroenteral ulcer. The results of the COX regression models are available in Table 2 and Supplementary Content 1.

**Table 2:** COX regression modeling for identification of risk factors for starting PPI treatment following L-RYGB and L-SG.

	HR^a^	95% CI	*P*
Surgery			
L-RYGB	1	1	
L-SG	7.06	6.42–7.77	**<0.0001**
Sex			
Male	1	1	
Female	1.29	1.20–1.39	**<0.0001**
Age (years)			
<30	1	1	
30–39	1.33	1.17–1.51	<**0.0001**
40–49	1.76	1.56–1.99	<**0.0001**
50–59	2.36	2.08–2.68	<**0.0001**
>60	2.35	2.96–2.81	<**0.0001**
Charlson comorbidity index			
0	1	1	
1	1.40	1.30–1.52	**<0.0001**
2	1.63	1.44–1.85	<**0.0001**
≥3	1.60	1.43–1.79	<**0.0001**
Preoperative treatment with PPI	3.10	2.65-3.62	**<0.0001**
Postoperative gastroenteral ulcer	3.97	3.62-4.36	**<0.0001**

*PPI* proton pump inhibitor, *L-RYGB* laparoscopic roux-en-y gastric bypass, *L-SG* laparoscopic sleeve gastrectomy, *HR* hazard ratio, *CI* confidence interval.

^a^Adjusted for surgery, sex, age, Charlson Comorbidity Index, preoperative treatment with PPI and postoperative gastroenteral ulcer.

Bold values are the values where the p-value is lower than 0.05.

### Postoperative continuous PPI treatment

**Fig. 3:**
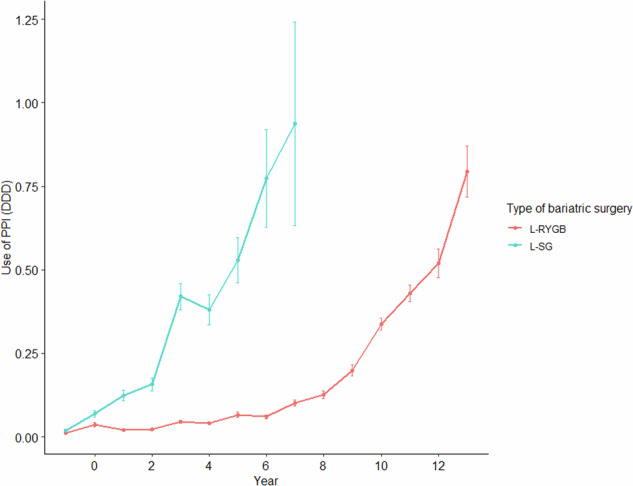
Use of proton pump inhibitors after laparoscopic Roux-en-Y gastric bypass and sleeve gastrectomy.

Overall, the PPI use increased following bariatric surgery, with a tendency to increase more prominently after L-SG than L-RYGB. By seventh year, the mean use increased from 0.05 to 0.94 DDD after L-SG, compared with an increase from 0.02 to 0.08 DDD after L-RYGB (see Fig. 3). Among patients receiving preoperative PPI treatment, usage decreased from 1.6 DDD preoperatively to 0.3–0.8 DDD postoperatively and remained relatively constant throughout the follow up. The continuous use of PPI was more frequent after L-SG versus L-RYGB, 2373 (15%) versus 421 (25%) patients, respectively. The risk was significantly higher after L-SG in a logistic regression model adjusted for sex, age, preoperative CCI, preoperative PPI treatment and postoperative occurrence of gastroenteric ulcer (OR 1.45, 95% CI 1.36–1.54). In a subgroup analysis including 11,494 patients furtherly adjusted for BMI, smoking, marital status, occupational status and educational level, the heightened risk persisted (see Supplementary Content 2). Risk factors for continuous PPI treatment additionally included female sex, age <30 or >40, smoking, CCI ≥ 3, intermediate educational level, retirement, preoperative PPI treatment and incidence of postoperative gastroenteral ulcer. Results of the logistic regression models are available in Table 3 and Supplementary Content 2. Respectively, 30% and 22% of patients undergoing L-RYGB and L-SG initiated treatment with ulcerogenic pharmaceuticals postoperatively. The time-dependent risk of initiating treatment with ulcerogenic pharmaceuticals was significantly higher after L-SG compared with L-RYGB (*p* < 0.0001).

**Table 3:** Logistic regression modeling for identification of risk factors for continuous PPI treatment following L-RYGB and L-SG.

	OR^a^	95% CI	*P*
Surgery			
L-RYGB	1	1	
L-SG	1.45	1.36–1.54	**<0.0001**
Sex			
Male	1	1	
Female	1.19	1.13–1.25	**<0.0001**
Age (years)			
<30	1	1	
30–39	0.78	0.72–0.85	<**0.0001**
40–49	1.15	1.06–1.24	**0.0005**
50–59	1.59	1.46–1.73	<**0.0001**
>60	1.48	1.25–1.74	<**0.0001**
Charlson comorbidity index			
0	1	1	
1	0.99	0.91–1.08	0.798
2	1.13	0.99–1.29	0.063
≥3	1.33	1.18–1.49	**<0.0001**
Preoperative treatment with PPI	1.77	1.40–2.23	**<0.0001**
Postoperative gastroenteral ulcer	6.32	5.43–7.35	**<0.0001**

*PPI* proton pump inhibitor, *L-RYGB* laparoscopic roux-en-y gastric bypass, *L-SG* laparoscopic sleeve gastrectomy, *OR* odds ratio, *CI* confidence interval.

^a^Adjusted for surgery, sex, age, Charlson Comorbidity Index, preoperative treatment with PPI and postoperative gastroenteral ulcer.

Bold values are the values where the p-value is lower than 0.05.

### Patients diagnosed with GERD

Postoperatively, 9556 gastroscopies were performed among 4675 (26%) unique patients. Of those undergoing gastroscopy, 5% were diagnosed GERD, corresponding to 1% of the study population. Following L-SG, 11% of the patients undergoing a gastroscopy were diagnosed with GERD, compared with 4% after L-RYGB. Assessment with 24 h pH measurement was conducted in 74 patients, and 12 were diagnosed with GERD. After L-SG, 27% of the patients assessed with 24 h pH measurement were diagnosed with GERD, compared with 3% after L-RYGB. Revisional procedures were performed in 99 patients, with significantly more L-SG patients undergoing revisional surgery (*p* < 0.0001). Five patients underwent anti-reflux surgery which consisted of modified laparoscopic cruroplasty. A composite of 26 (2%) L-SG patients were diagnosed with GERD after relevant investigative procedures, compared with 192 (1%) of the L-RYGB patients. The risk of being diagnosed with GERD over time is presented in Fig. 2B, indicating a significantly increased risk after L-SG compared with L-RYGB (*p* = 0.0001). In a multivariate COX regression adjusted for sex, age, preoperative CCI and PPI treatment, the higher risk after L-SG persisted, HR 1.93 (95% CI 1.27–2.93).

## Discussion

This nationwide cohort study provides long-term data on use of PPI after bariatric surgery. Patients after L-SG both had a significantly higher risk of initiating and continuing PPI treatment, and utilized higher dosages, which were increasing throughout the follow up period. When evaluating diagnosis codes in relation to investigative procedures, L-SG patients were significantly overrepresented among those diagnosed with GERD.

Limited studies have longitudinally investigated PPI use after bariatric surgery. Our findings for the first four years align with a French register-based study [27], but our study revealed a continuous increase resulting in a seven-fold higher risk of de-novo and persistent PPI use after L-SG. The increase in PPI among patients after L-RYGB can in part be explained by gastroenteric ulcers (OR 6.32, 95% CI 5.43–7.35). However, this is not a probable explanation after L-SG after which the risk of gastroenteric ulcers is negligible [28, 29]. The heightened risk of GERD after L-SG aligns with previous studies reporting a pooled rate of de-novo reflux of 20% and a worsening of existing reflux among 19% [30]. However, a recent systematic review presents lower rates of de-novo GERD after L-SG, suggesting satisfactory control of postoperative GERD and supporting L-SG as a viable bariatric procedure, even in patients with obesity and pre-existing GERD [31]. The rates from previous studies are considerably higher compared with the present study. Discrepancies in rates may be a result of differences in definitions, and the definition in our study is likely to have captured only the severe cases requiring evaluation at specialized centers [31].

The increased risk of GERD has prompted concerns from the International Federation for Obesity (IFSO), leading to updated recommendations for systematic pre- and postoperative endoscopic control [32]. While several studies suggest a high risk of Barrett’s esophagus after L-SG, the possible effect of PPI on the risk of development of high-grade dysplasia or esophageal adenocarcinoma has not been established [33–35]. Furthermore, no significant increase in the risk of high-grade dysplasia or esophageal adenocarcinoma has been established.

Study limitations, inherent to the availability of PPI, include the possibility of acquiring low doses over-the counter (equivalent to 10 mg omeprazole). However, latest national evaluation found that only 3% of the PPI consumption was acquired over-the counter [36]. Use of other antacids such as H2-recepter antagonist have not been included as these are seldom used in Denmark [37]. The primary indication for PPI in this population is hypothesized to be GERD. Other considerable indications are prophylactic use in case of simultaneous ulcerogenic medications, as a treatment of gastroenteric ulcers and on a trial-basis in case of vague symptoms. The Cox regression model was adjusted for the postoperative occurrence of gastrointestinal ulcers. Although more patients appeared to have started treatment with ulcerogenic pharmaceuticals after L-SG, adequate adjustments were not possible as these are bought over-the-counter in Denmark. Limitations linked with the register-based design include the risk of wrongful registration of NPR codes for definition of GERD and the lack of patient related outcome measures, potentially leading underestimation of the incidence of GERD [38]. Patients with symptomatic GERD treated at general practitioners were not included, and patients without subsequent outpatient evaluation might not be correctly diagnosed. In contrast, non-specific symptoms could wrongfully be interpreted as GERD leading to overestimation of the incidence. But as the definition relied on diagnosis codes related to specific investigative procedures, the risk of misclassification or overestimation was considered minimal. However, despite access to public healthcare, regional differences in the availability and use of endoscopy, 24 h pH measurement and the decisions to do revisional bariatric and anti-reflux surgery might exist. Overall, we hypothesize a considerable underestimation of the incidence of GERD. Finally, the extended follow up after L-RYGB compared with L-SG introduced a limitation. Consequently, the outcomes regarding evaluation of continuous PPI treatment for L-SG after the median follow up of four years could be less accurate compared with L-RYGB. However, as multivariate analyses were conducted time-independently, the significance of this limitation is deemed negligible. Nevertheless, an even more extended follow-up period for both L-RYGB and L-SG are likely required before the PPI use reaches steady state (see Fig. 2).

While external validity may vary due to endemic factors, differing definitions of GERD and availability of PPI, the large and nationwide cohort with complete data from Danish health registries enhances its generalizability to similar international cohorts [2].

In conclusion, the risk of initiating and continuing PPI treatment was significantly higher after L-SG compared with L-RYGB. A continuous increase in the utilization of PPI was observed after both procedures. The risk of GERD diagnosis was also significantly higher after L-SG compared with L-RYGB. Future research should focus on severe consequences such as Barrett’s esophagus and evaluation of surgical options for GERD treatment, particularly after L-SG. Incorporating patient related outcomes measures would contribute with valuable insights into symptom extent and severity, aiding in the identification of suitable patients for L-RYGB and L-SG.

## Supplementary information

Supplementary material 1

Supplementary material 2
