# Supplementary material for: Use of proton pump inhibitors after laparoscopic gastric bypass and sleeve gastrectomy: a nationwide register-based cohort study
Source: Int J Obes (Lond). 2024 Jul 23;48(11):1613–9. doi: 10.1038/s41366-024-01593-5 (PMC11502493; doi:10.1038/s41366-024-01593-5)
Supplement: Supplementary file 1 — Supplementary material 1 [file 41366_2024_1593_MOESM1_ESM.docx]

Table 1. COX regression modelling for identification of risk factors for starting PPI treatment following L-RYGB and L-SG, subgroup analysis with inclusion of 11 494 patients.

|  | HR ^a^ | 95% CI | P |
| --- | --- | --- | --- |
| **Surgery** L-RYGB L-SG | 1  5.74 | 1  5.17-6.39 | **<0.0001** |
| **Sex** Male  Female | 1  1.19 | 1  1.09-1.30 | **<0.0001** |
| **Age** (years)  < 30  30-39  40-49  50-59  > 60 | 1  1.32  1.77  2.37  2.05 | 1  1.11-1.57  1.50-2.10  1.99-2.82  1.62-2.60 | **0.001**  **<0.0001**  **<0.0001**  **<0.0001** |
| **BMI** (kg/m^2^) n = 13,552  < 39.9  40-44.9  45-49.9  50-54.9  > 60 | 1  0.90  0.97  0.92  0.94 | 1  0.81-0.99  0.86-1.08  0.80-1.05  0.80-1.11 | **0.040**  0.536  0.229  0.942 |
| **Smoking status** n = 13,233  Yes  Never or previously | 1.21  1 | 1.11-1.33  1 | **<0.0001** |
| **Charlson Comorbidity Index**  0  1  2  ≥3 | 1  1.26  1.50  1.38 | 1 1.15-1.39 1.30-1.73 1.21-1.58 | **<0.0001 <0.0001 <0.0001** |
| **Preoperative treatment with PPI** | 3.74 | 2.55-3.57 | **<0.0001** |
| **Postoperative gastroenteral ulcer** | 3.40 | 3.02-3.83 | **<0.0001** |
| **Marital status** n = 17,618  Married or cohabiting  Single | 1  1.06 | 1  0.97-1-15 | 0.200 |
| **Occupational status** n = 17,726  Employed  Unemployed  Retired | 1  1.52  1.57 | 1  1.37-1-68 1.40-1-75 | **<0.0001 <0.0001** |
| **Educational level** n = 17,433  Primary (up to 10^th^ grade)  High-school and vocational education  Short higher education, bachelor and equivalent  Master or equivalent including PhD grade | 1  1.47  1.36  0.99 | 1 0.70-3.09 0.64-2.88  0.44-2.24 | 0.312 0.419  0.987 |

^a^ adjusted for surgery, sex, age, BMI, smoking, Charlson Comorbidity Index, marital status, occupational status, educational level, preoperative treatment with PPI and postoperative gastroenteral ulcer.
Abbreviations: PPI, proton pump inhibitor; L-RYGB, laparoscopic roux-en-y gastric bypass; L-SG, laparoscopic sleeve gastrectomy; HR, hazard ratio; CI, confidence interval; BMI, body mass index.
