# Supplementary material for: Use of proton pump inhibitors after laparoscopic gastric bypass and sleeve gastrectomy: a nationwide register-based cohort study
Source: Int J Obes (Lond). 2024 Jul 23;48(11):1613–9. doi: 10.1038/s41366-024-01593-5 (PMC11502493; doi:10.1038/s41366-024-01593-5)
Supplement: Supplementary file 2 — Supplementary material 2 [file 41366_2024_1593_MOESM2_ESM.docx]

Table 2. Logistic regression modelling for identification of risk factors for continuous PPI treatment following L-RYGB and L-SG, subgroup analysis with inclusion of 11 494 patients.

|  | OR ^a^ | 95% CI | P |
| --- | --- | --- | --- |
| **Surgery** L-RYGB L-SG | 1  1.44 | 1  1.35-1.55 | **<0.0001** |
| **Sex** Male  Female | 1  1.15 | 1  1.08-1.22 | **<0.0001** |
| **Age** (years)  < 30  30-39  40-49  50-59  > 60 | 1  0.78  1.16  1.59  1.38 | 1  0.70-0.87  1.05-1.27  1.43-1.77  1.14-1.68 | **<0.0001**  **0.003**  **<0.0001**  **0.001** |
| **BMI** (kg/m^2^) n = 13,552  < 39.9  40-44.9  45-49.9  50-54.9  > 60 | 1  0.99  0.98  0.90  1.10 | 1  0.91-1.08  0.88-1.08  0.79-1.03  0.95-1.29 | 0.858  0.655  0.113  0.211 |
| **Smoking status** n = 13,233  Yes  Never or previously | 1.40  1 | 1.24-1.58  1 | **<0.0001** |
| **Charlson Comorbidity Index**  0  1  2  ≥3 | 1  0.99  1.16  1.17 | 1  0.89-1.10  0.99-1.35  1.02-1.35 | 0.862  0.056  **0.029** |
| **Preoperative treatment with PPI** | 1.90 | 1.47-2.44 | **<0.0001** |
| **Postoperative gastroenteral ulcer** | 5.60 | 4.63-6.79 | **<0.0001** |
| **Marital status** n = 17,618  Married or cohabiting  Single | 1.08  1 | 0.96-1.21  1 | 0.185 |
| **Occupational status** n = 17,726  Employed  Unemployed  Retired | 1  1.04  1.38 | 1  0.94-1.15  1.24-1.52 | 0.417  **<0.0001** |
| **Educational level** n = 17,433  Primary (up to 10^th^ grade)  High-school and vocational education  Short higher education, bachelor and equivalent  Master or equivalent including PhD grade | 1  1.72  1.46  0.82 | 1  1.24-2.39  1.04-2.04  0.52-1.31 | **0.001**  **0.029**  0.415 |

^a^ Adjusted for surgery, sex, age, BMI, smoking, Charlson Comorbidity Index, marital status, occupational status, educational level, preoperative treatment with PPI and postoperative gastroenteral ulcer.
Abbreviations: PPI, proton pump inhibitor; L-RYGB, laparoscopic roux-en-y gastric bypass; L-SG, laparoscopic sleeve gastrectomy; HR, hazard ratio; CI, confidence interval; BMI, body mass index.
